# Concomitant taurine exposure counteracts ethanol-induced changes in locomotor and anxiety-like responses in zebrafish

**DOI:** 10.1007/s00213-019-05410-0

**Published:** 2019-11-30

**Authors:** Barbara D. Fontana, Tamie Duarte, Talise E. Müller, Julia Canzian, Paola R. Ziani, Nathana J. Mezzomo, Matthew O. Parker, Denis B. Rosemberg

**Affiliations:** 1 Brain and Behaviour Laboratory, School of Pharmacy and Biomedical Sciences, University of Portsmouth, England, UK; 2 Laboratory of Experimental Neuropsychobiology, Department of Biochemistry and Molecular Biology, Natural and Exact Sciences Center, Federal University of Santa Maria, 1000 Roraima Avenue, Santa Maria, RS 97105-900 Brazil; 3 Graduate Program in Biological Sciences: Toxicological Biochemistry, Federal University of Santa Maria, 1000 Roraima Avenue, Santa Maria, RS 97105-900 Brazil; 4 Graduate Program in Pharmacology, Federal University of Santa Maria, 1000 Roraima Avenue, Santa Maria, RS 97105-900 Brazil; 5 The International Zebrafish Neuroscience Research Consortium (ZNRC), 309 Palmer Court, Slidell, LA 70458 USA

**Keywords:** Alcohol, Energy drink, Anxiety, Exploration, Zebrafish

## Abstract

Taurine (TAU) is a β-amino sulfonic acid with pleiotropic roles in the brain, including the neuromodulatory activity via GABAergic and glycinergic agonism. This molecule is found at high concentrations in energy drinks and is often mixed with alcohol in beverages. Although TAU has a neuroprotective role in the brain, the putative risks of mixing TAU and EtOH are not fully understood. Here, we investigated whether TAU modulates locomotor and anxiety-like behavior in adult zebrafish by using the novel tank and light-dark tests following acute EtOH exposure at anxiogenic and anxiolytic concentrations. Zebrafish were individually exposed to water (control), TAU (42, 150, and 400 mg/L), and EtOH (0.25% (v/v) and 1% (v/v)) both independently and cotreated for 1 h. EtOH 0.25% and TAU produced U-shape anxiolytic-like behavior in the light-dark test, TAU 42 and 400 positively modulated EtOH effects, and TAU 150 exerted a protective effect. All TAU concentrations counteracted EtOH 1%-induced locomotion impairment, as well as the anxiogenic-like behavior. Finally, all TAU concentrations when given independently or cotreated with EtOH 0.25% and 1% decreased the risk assessment of the lit compartment. Principal component analyses revealed that exploration and anxiety-like responses were the main behaviors that contribute to the effects of TAU and EtOH. Overall, we demonstrate that TAU differently modulates EtOH-induced anxiolytic- and anxiogenic-like behaviors depending on the concentration, suggesting a complex mechanism underlying TAU and EtOH interactions.

## Introduction

Ethanol (EtOH) is one of the most widely consumed psychoactive drugs worldwide (Degenhardt et al. 2008). EtOH acutely promotes a biphasic effect, where low to moderate doses are stimulant and higher doses induce depressant effects (Addicott et al. 2007). In the brain, the mechanisms underlying the effects of EtOH include neuroinflammation, glial effects, and impaired neuromodulation (Lasek 2016; Montesinos et al. 2016). Furthermore, alcohol has stress-reducing properties, and the chronic use can escalate into alcohol use disorders (Cooper et al. 1995; Schroder and Perrine 2007), which frequently comorbid with anxiety-related disorders (Grant et al. 2004; Kushner et al. 1990). Patients with mood and anxiety disorders use substances to cope with the negative symptoms associated with the diseases, thereby corroborating a high correlation between anxiety and alcohol use disorder. Then, increased alcohol consumption for a long period can facilitate substance use disorder (Turner et al. 2018).

Taurine (TAU) is a β-amino sulfonic acid found at high concentrations in energy drinks (Heckman et al., 2010) and is commonly mixed with alcohol in several popular beverages (Ferreira et al. 2004a; Ferreira et al. 2004b; Marczinski and Fillmore 2014). This molecule acts as osmotic regulator (Schaffer et al. 2010), membrane stabilizer (Lambert et al. 2015), antioxidant (Lerdweeraphon et al. 2013), and inhibitory neuromodulator (Wu and Prentice 2010), as well as in the maintenance of intracellular calcium metabolism (Foos and Wu 2002). Although most studies do not investigate the direct interaction of TAU/EtOH, the combined used of EtOH and energy drinks is associated to major health problems. Thus, the combined use of alcohol and energy drinks increases the number and time of drinks consumed, dehydration, and more severe and prolonged hangovers, leading to an increase number of alcohol poisoning reports (Patrick et al. 2014; Snipes and Benotsch 2013).

TAU modulatory activity has been shown to regulate distinct behaviors, causing a negative impact when associated with drugs of abuse depending on the concentration (Fontana et al. 2016; Fontana et al. 2018; Mezzomo et al. 2018). For example, high concentrations of TAU associated to alcohol can increase aggressive behavior (Fontana et al. 2016) and decrease seeking for conspecifics (Fontana et al. 2018) in zebrafish. The hypothesis underlying the negative interaction of TAU with alcohol suggests an involvement of GABA_A_, where both molecules have an agonist role (Huxtable 1992; Olsen and Liang 2017; Wallner and Olsen 2008). Because the GABAergic system is involved in anxiety-related disorders (Lydiard 2003; Tasan et al. 2011) and considering that EtOH and TAU are concomitantly found in alcohol beverages, the study of their association is relevant to better understand how the interaction between these molecule can affect anxiety-related phenotypes in vertebrate systems.

The zebrafish (*Danio rerio*) is a suitable model species for modeling human diseases due to its high genetic and physiological homology, as well its well-characterized behavioral repertoire (Fontana et al. 2018; Kalueff et al. 2013; Parker et al. 2012; Stewart et al. 2015). Although EtOH and TAU alone modulate locomotion and anxiety-like behaviors in zebrafish (Gerlai et al. 2000; Mezzomo et al. 2016), the influence of their cotreatment in anxiety-like phenotypes has been poorly explored. Thus, we aimed to evaluate the role of TAU and EtOH (low and high concentrations) in modulating anxiety-like responses in zebrafish using two behavioral tests (novel tank and light-dark tests). Using the principal component analysis (PCA), we also assessed which parameters have more impact in the behavioral changes induced by TAU and EtOH concomitant exposure.

## Materials and methods

### Animals

Adult zebrafish (*Danio rerio*) (50:50 male: female ratio; wild-type; short fin phenotype) were obtained from a commercial supplier (Hobby Aquários, RS, Brazil). Animals were maintained for 2 weeks in 50 L tanks filled with non-chlorinated water at a density of two animals per liter under controlled temperature (26 ± 2 °C), pH (7.0–7.5), and photoperiod cycle (14:10 light-dark; lights on at 7:00 a.m.). Fish were fed twice daily with commercial flake food (Alcon Basic®, Alcon, Brazil). All behavioral tests were performed during the same time each day (between 10:00 a.m. and 4:00 p.m.) immediately after TAU or/and EtOH exposure. Importantly, two independent batches were used for the experiments, and each animal was tested in a single apparatus (novel tank or light-dark test) to avoid stress caused by subsequent manipulations. The tank water was changed after each trial to remove potential cues that influence behavior. Both housing and experimental conditions were in accordance to the National Institute of Health Guide for Care and Use of Laboratory Animals. All protocols used in this study were previously approved by the Ethics Commission on Animal Use of the Federal University of Santa Maria (process number 026/2014).

### Treatments

To assess the effects of TAU (Sigma, St. Louis, MO, USA) and EtOH (Merck, Darmstadt, Germany) association, fish were exposed individually in 500 mL beakers for 1 h. The following groups were tested: control (non-chlorinated water), TAU alone at 42 mg/L (TAU 42), 150 mg/L (TAU 150), and 400 mg/L (TAU 400) and EtOH 0.25% (v/v) alone or cotreated with TAU (TAU 42/EtOH 0.25%, TAU 150/EtOH 0.25%, and TAU 400/EtOH 0.25%) and EtOH 1% (v/v) alone or cotreated with TAU (TAU 42/EtOH 1%, TAU 150/EtOH 1%, and TAU 400/EtOH 1%). TAU concentrations were based on previous reports, which showed anxiolytic-like properties, as well as a significant modulatory role on EtOH-induced behaviors (Fontana et al. 2016; Mezzomo et al. 2016; Rosemberg et al. 2012). In order to assess both anxiolytic- and depressant-like effects of alcohol in zebrafish, two concentrations (0.25% v/v and 1% v/v) were chosen as described elsewhere (Gerlai et al. 2000). A total of 288 fish were used for the experiments.

### Novel tank diving test

The novel tank diving test is one of the most used apparatus to assess locomotor and anxiety-like phenotypes, showing a high sensitivity to anxiolytic and anxiogenic manipulations (Egan et al. 2009; Kalueff et al. 2013; Levin et al. 2007; Maximino et al. 2010b; Mezzomo et al. 2016; Wong et al. 2010). After the exposure period, animals (*n =* 10–12) were placed individually in the apparatus (25 cm length x 15 cm height x 6 cm width). Behaviors were recorded using a webcam for 6 min based on previous studies, where animals gradually spend more time in the top portion of a tank over the first 6 min of the test (Blaser and Rosemberg 2012; Egan et al. 2009; Mezzomo et al. 2016; Rosemberg et al. 2012; Wong et al. 2010). Data were analyzed in an automated fashion using a video-tracking software (ANY-maze^TM^, Stoelting CO, USA) at a rate of 30 frames/s. The tank was divided in three virtual areas (bottom, middle, and top) to provide a detailed evaluation of the vertical activity. The following endpoints were measured: total distance traveled, absolute turn angle, latency to enter the top area, time spent in top area, transitions to top area, and immobility.

### Light-dark test

The light-dark test is a complementary task to assess anxiety-like responses, which explores the natural tendency of adult zebrafish to avoid brightly lit environments (Blaser and Rosemberg 2012; Maximino et al. 2010a; Maximino et al. 2010b; Rosemberg et al. 2011). This test was performed in experimental tanks (30 cm length x 10 cm width x 15 cm height) divided into two equally sized partitions using black or white self-adhesive film to cover the walls and floor. After the treatments, zebrafish (*n =* 10–12) were gently placed in the white partition of the tank, and their behaviors were quantified for 6 min using the ANY-maze^TM^ software (Stoelting CO, USA) at a rate of 30 frames/s. The following endpoints were determined: time spent in lit area, transitions to lit area, latency to enter the dark area, and number of risk assessment episodes. Risk assessment was assessed manually by three trained observers blinded to the experimental condition (inter-rater reliability > 0.90) and defined as a fast (>1 s) entry in the white compartment followed by reentry in the black compartment, or as a partial entry in the lit area (Kalueff et al. 2013; Mezzomo et al. 2016).

### Statistics

Data were checked for normality and homogeneity of variances via the Kolmogorov-Smirnov and Bartlett’s tests, respectively. Data were expressed as means ± standard error of the mean (S.E.M) and further analyzed by two-way or three-way ANOVA followed by Tukey’s multiple comparisons test. Given the asymmetric data distribution of the latencies to enter to the top/dark areas, the respective parameters were expressed as median ± interquartile range and analyzed by Kruskal-Wallis test followed by Dunn’s multiple comparison test. PCA was performed to evaluate the potential correlation between variables of the novel tank diving task and the light-dark test, separately. The component matrix was subjected to Varimax rotation with Kaiser normalization. Components (or factors) with eigenvalue lower than 1 were disregarded, and measures with loadings greater than 0.4 were retained. The first principal component (PC1) explains the largest percentage of data variance. Data were analyzed in SPSS 19 (IBM SPSS Statistics, version 19). Post hoc analyses were performed using Tukey’s multiple comparisons test when appropriate. The significance was set at *p* ≤ 0.05.

## Results

### TAU counteracts EtOH-induced locomotor deficits and anxiogenic-like behavior

**Fig. 1:**
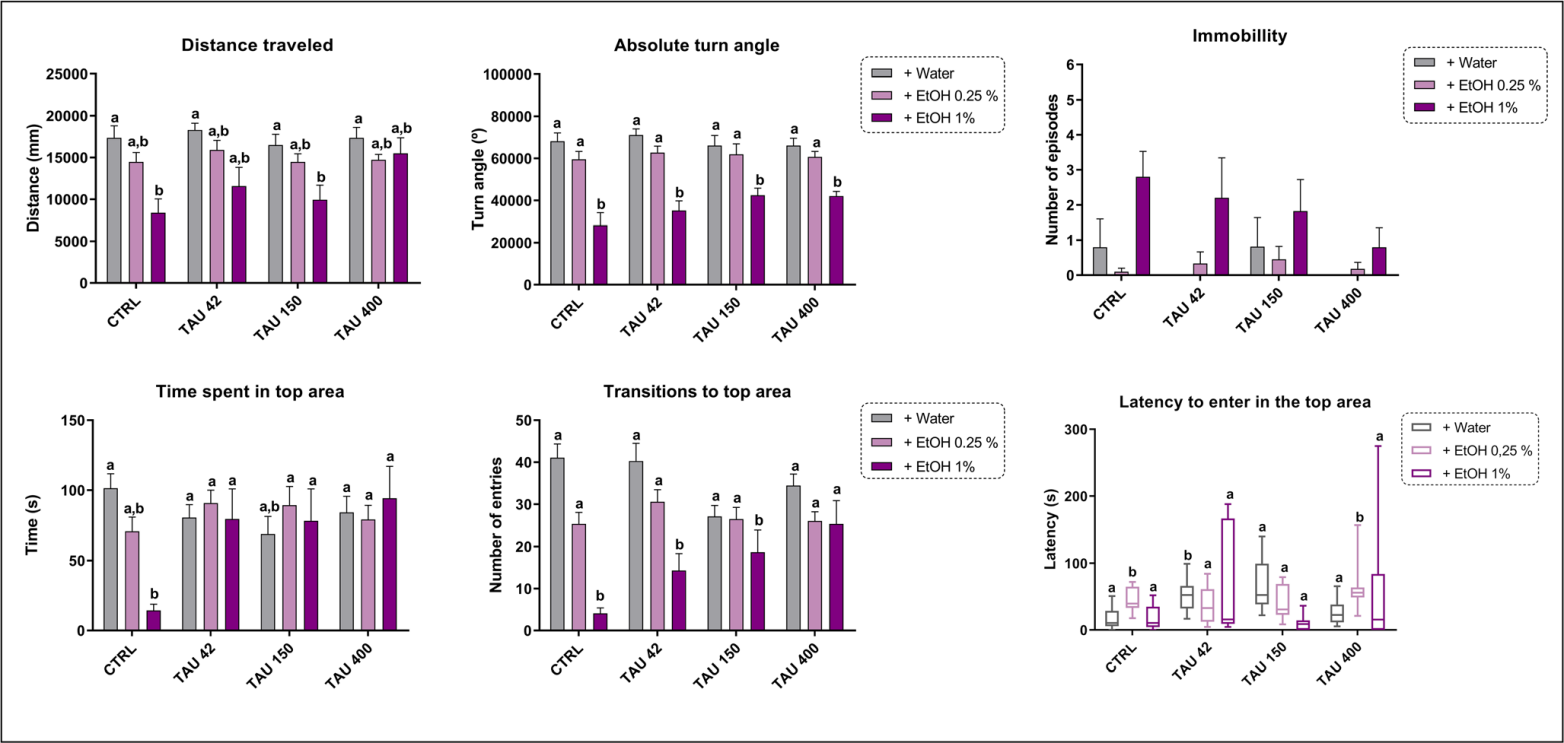
Effects of TAU and/or EtOH in the novel tank task. Data were represented as mean ± S.E.M. and analyzed by two-way ANOVA, followed by Tukey’s multiple comparison test. Different letters indicate statistical differences between groups (*p* < 0.05, *n =* 12 per group)

Figure 1 shows the effects of TAU and EtOH in the novel tank task. Two-way ANOVA yielded a significant EtOH effect on distance traveled (*F*_(2,116)_ = 18.51, *p <* 0.0001), absolute turn angle (*F*_(2,116)_ = 67.65, *p <* 0.0001), and transitions to top area (*F*_(2,116)_ = 33.04, *p <* 0.0001). Furthermore, a significant TAU × EtOH interaction was observed for the time spent in top (*F*_(6,116)_ = 3.070, *p =* 0.0081) and transitions to top area (*F*_(6,116)_ = 4.090, *p =* 0.0009). TAU 42 (*p* < 0.005) and 150 mg/L (*p* < 0.005) alone increased the latency to enter the top area, while EtOH 0.25% alone did not show significant differences in the novel tank test when compared with control. Regarding the effects of EtOH 1%, only EtOH 1% and TAU 150/EtOH 1% groups showed a reduced distance traveled. Additionally, EtOH 1% alone and all TAU-associated groups showed decreased absolute turn angle and transitions to top area, except TAU 400/EtOH 1%, which revealed only a decreased absolute turn angle. Animals exposed to 1% EtOH alone spent less time in top area, but this effect was abolished by all TAU concentrations. Temporal analyses of the time spent in the top area showed that EtOH significantly affected the diving responses across time (*F*_(10,116)_ = 4.634, *p* < 0.0001). Finally, a significant interaction of time × TAU × EtOH was observed (*F*_(30,116)_ = 1.647, *p* = 0.017) (data not shown).

### TAU induces biphasic reduction of anxiolytic-like behavior

**Fig. 2:**
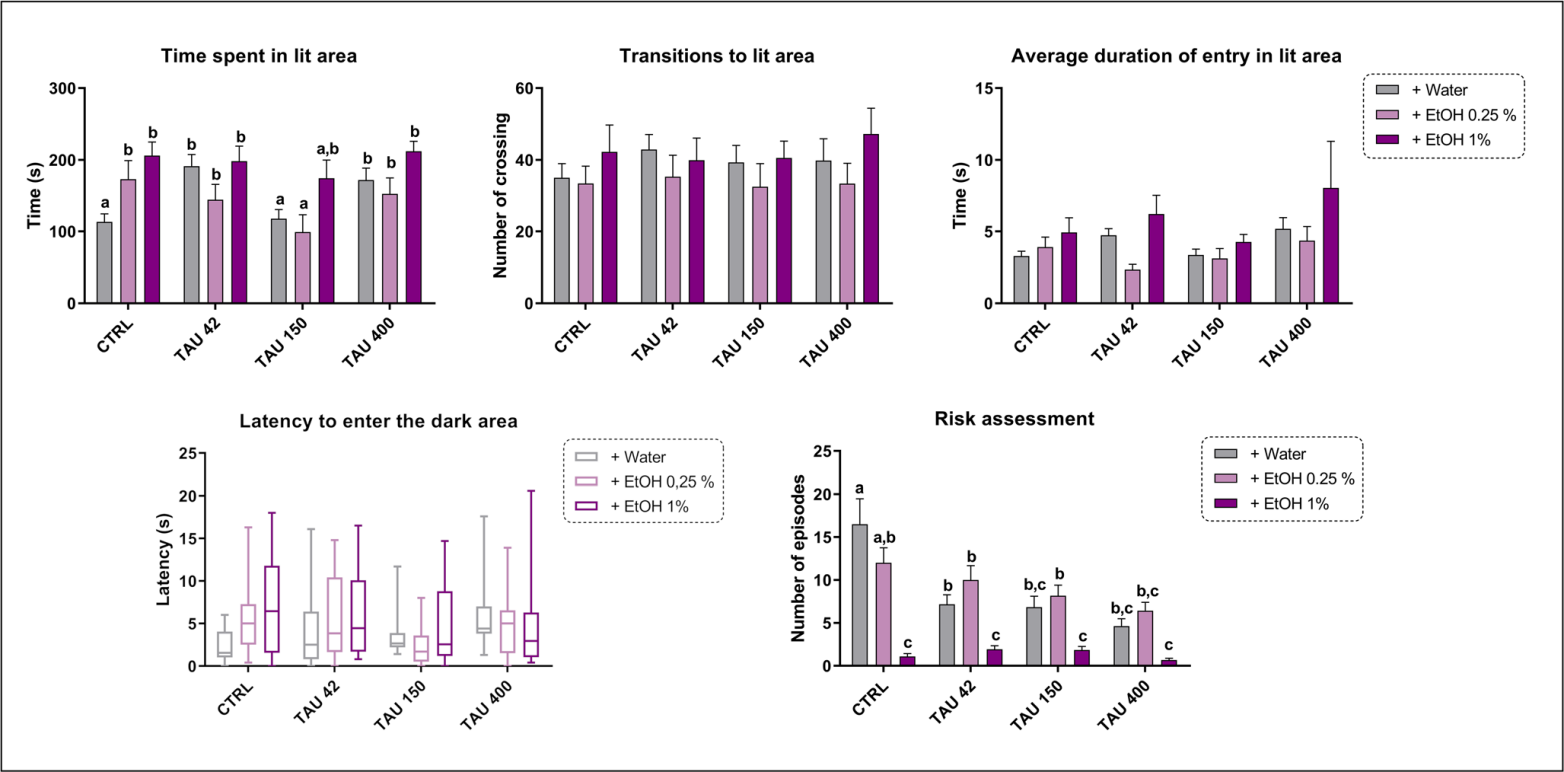
Effects of TAU and/or EtOH in the light-dark test. Data were represented as mean ± S.E.M. and analyzed by two-way ANOVA, followed by Tukey’s multiple comparison test. Different letters indicate statistical differences between groups (*p* < 0.05, *n =* 12 per group)

Figure 2 shows the behavioral responses of zebrafish in the light-dark test. Significant TAU effects were observed in the time spent in lit area (*F*_(3,122)_ = 2.404, *p =* 0.0440) and risk assessment episodes (*F*_(3,122)_ = 11.43, *p <* 0.0001). Significant EtOH effects were observed for the time spent in lit area (*F*_(2,122)_ = 12.99, *p <* 0.0001), average duration of entry in the lit area (*F*_(2,122)_ = 4.233, *p =* 0.0167), and risk assessment episodes (*F*_(2,122)_ = 48.57, *p <* 0.0001). Furthermore, a TAU × EtOH interaction was observed for both time spent in lit area (*F*_(6,122)_ = 2.404, *p =* 0.0313) and risk assessment episodes (*F*_(6,122)_ = 4.403, *p =* 0.0004). The effects of TAU alone in the light-dark test revealed that TAU 42 and TAU 400 groups spent more time in the lit area. Regarding EtOH 0.25% effects, EtOH alone increased the time spent in lit area. Two-way ANOVA revealed that EtOH 1%, TAU42/EtOH 1%, and TAU400/EtOH 1% groups spent more time in the lit area. Additionally, TAU groups (alone or concomitantly exposed to alcohol) showed decreased number of risk assessments. Temporal analyses yielded a significant time × TAU × EtOH interaction (*F*_(30,122)_ = 2.431, *p* = 0.000), while only EtOH modulated the light-dark responses across time (*F*_(10,122)_ = 1.968, *p* = 0.034).

### Exploratory- and anxiety-related parameters are the main components involved in the behavioral responses measured

**Fig. 3:**
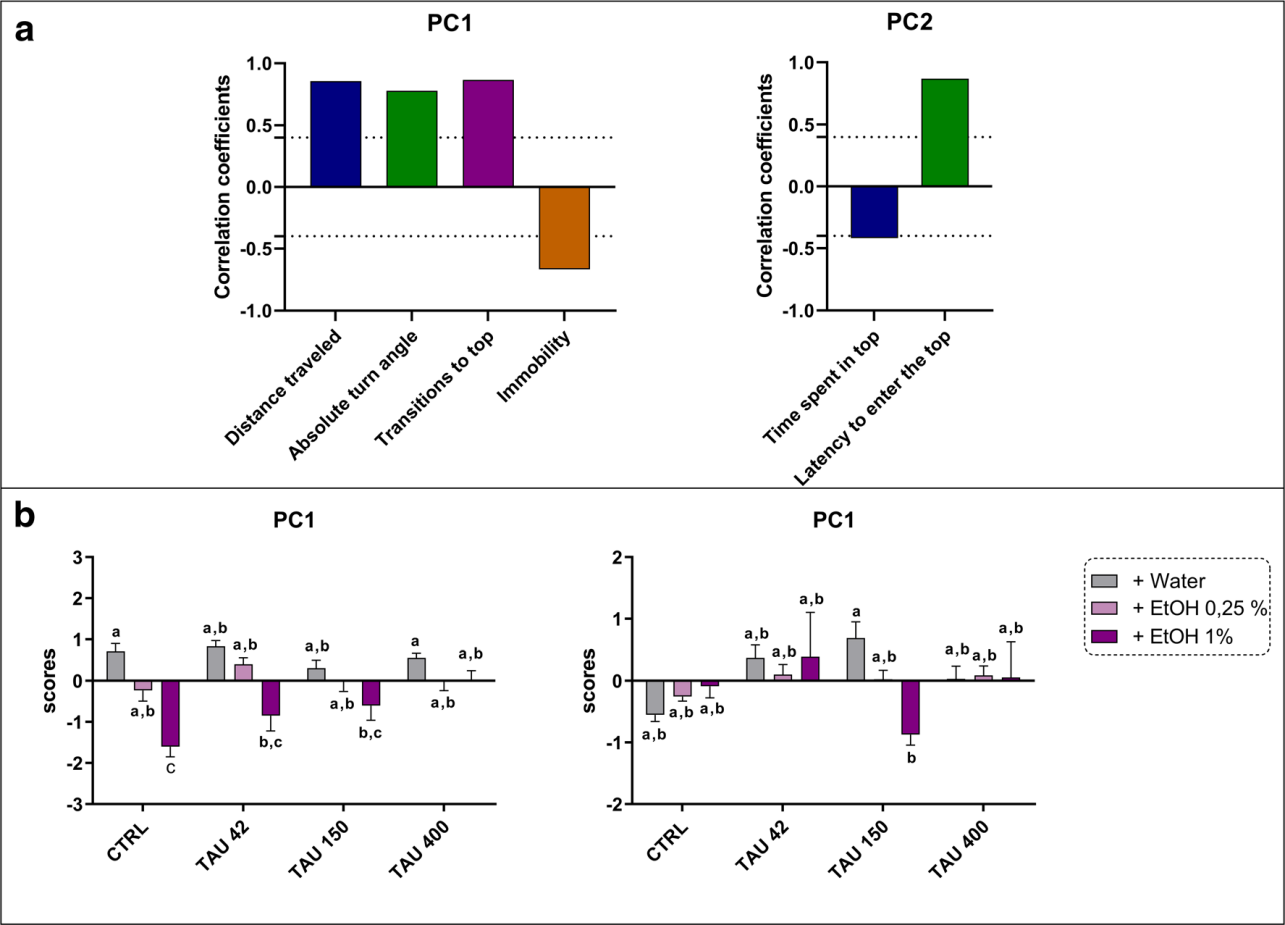
Principal component analysis (PCA) for behavioral endpoints measured in the novel tank test. (A) Correlation coefficients between the behavioral endpoints for each PC. Dashed lines represent cut-off points, and only loadings greater than 0.3 or smaller than −0.4 are depicted. (B) Comparison of the values of two PC with eigenvalues greater than 1. Data are expressed as mean ± SEM and analyzed by two-way ANOVA, followed by Tukey’s post hoc test. Distinct letters indicate statistical differences between experimental groups (*p* < 0.05, *n =* 12 per group)

**Fig. 4:**
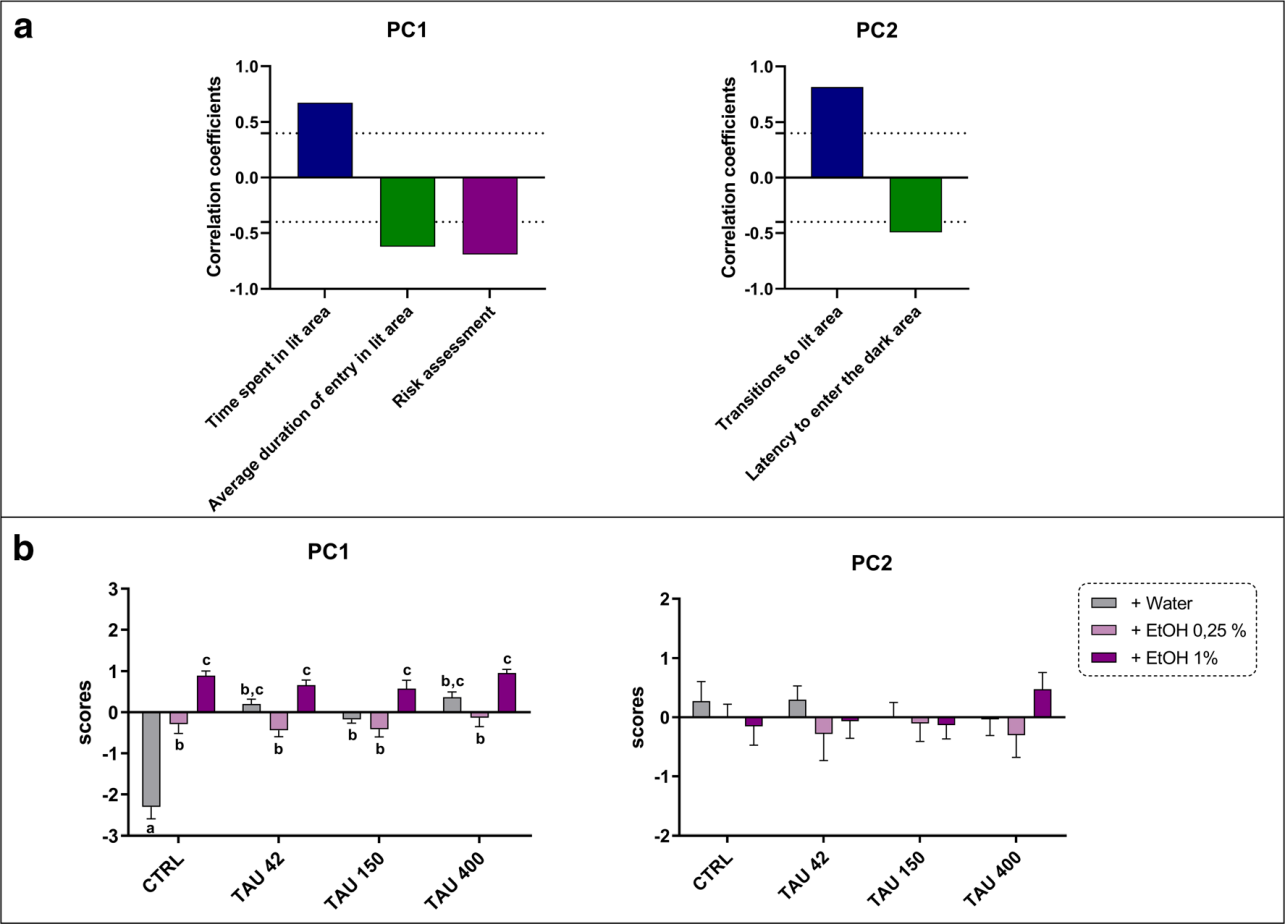
Principal component analysis (PCA) for behavioral endpoints measured in the light-dark test. (A) Correlation coefficients between the behavioral endpoints for each PC. Dashed lines represent cut-off points and only loadings greater than 0.3 or smaller than − 0.4 are depicted. (B) Comparison of the values of two PC with eigenvalues greater than 1. Data are expressed as mean ± SEM and analyzed by two-way ANOVA, followed by Tukey’s post hoc test. Distinct letters indicate statistical differences between experimental groups (*p* < 0.05, *n =* 12 per group)

Figure 3 and 4 show the main components driving the behavioral responses of zebrafish when exposed to TAU or/and EtOH in the novel tank and light-dark test, respectively. For novel tank and light-dark tests, the Kaiser-Meyer-Olkin measure of sampling adequacy were 0.700 and 0.512, respectively. Bartlett’s tests of sphericity were also significant for novel tank task (χ2 = 244.236, df = 15, *p* < 0.000) and light-dark test (χ2 = 36.883, df = 10, *p* < 0.0001).

In the novel tank task, PCA extracted two principal components which accounted for more than 63% of the total variance (Fig. 3A). PC1 was associated with locomotion and exploratory activity, with positive component loadings for distance traveled, angular velocity, transitions to top, and a negative component loading for immobility. Meanwhile, the PC2 was associated with anxiety-related parameters, with a negative component for time spent in top and a positive component for latency to enter in top area. Two-way ANOVA yielded significant effects of TAU × EtOH interaction (*F*_(6,116)_ = 3.332, *p* = 0.0044), TAU (*F*_(3,116)=_ 3.582, *p* = 0.0158), and EtOH (*F*_(2,116)=_ 33.20, *p* = 0.0001) for the PC1. Post hoc analysis showed that EtOH 1% significantly affect the PC1, showing reduced values when concomitantly exposed with TAU 400 mg/L. For the PC2, a significant TAU × EtOH interaction (*F*_(6,116)=_ 2.584, *p* = 0.0215) was observed, in which TAU 150 vs TAU 150 + EtOH 1% showed significant differences.

In the light-dark test, PCA extracted two principal components accounting for 60% of the total variance (Fig. 4A). PC1 was highly associated to anxiety-related parameters, in which the time spent in lit area was loaded as a positive component, while the average duration of entry in lit area and the risk assessment were loaded as negative components. PC2 was associated to exploratory patterns in the light-dark test, loading a positive component for transitions in lit area and a negative one for latency to enter the dark area. Significant TAU × EtOH interaction (*F*_(6,122)_ = 18.51, *p* = 0.0001), TAU (*F*_(3,122)=_ 16.80, p = 0.0001), and EtOH (*F*_(2,122)=_ 63.26, p = 0.0001) effects were observed for the PC1 analysis. Post hoc analysis yielded a significant increase of PC1 values for TAU, EtOH 0.25%, EtOH 1% alone, and TAU-associated groups when compared to the control (Fig. 4B).

## Discussion

In this study, we evaluated the concomitant effects of TAU- and EtOH-induced changes in anxiety-like behaviors. We have shown for the first time that TAU attenuates EtOH-induced locomotor and anxiogenic-like phenotypes in the novel tank test. Moreover, when associated with EtOH, TAU results in a U-shaped response in light-dark test, exerting an anxiolytic-like effect in the lowest and highest concentrations, with a preventive effect at 150 mg/L. Moreover, all TAU concentrations tested decreased the risk assessment to lit compartment.

EtOH is an anxiolytic molecule at lower concentrations. In humans, for example, alcohol may be consumed to counteract acute social anxiety, and this reinforcing effect can even increase future use with EtOH acting as a “social reward” (Goodman et al. 2018). The mechanisms underlying the biphasic effects of alcohol in anxiety-related behaviors include the antagonism of glutamatergic receptors and the increase in dopamine and serotonin (5-HT) levels (Alasmari et al. 2018; Deehan et al. 2016). Additionally, high EtOH concentrations positively modulate GABA_A_ and GABA_B_ receptors (Frye and Fincher 1996; Krystal et al. 2003; Olsen and Liang 2017). As observed previously (Gerlai et al. 2000), EtOH 0.25% showed anxiolytic-like effects in the light-dark test. Conversely, EtOH 1% altered exploration, showing anxiogenic-like responses in the novel tank test (Blaser and Penalosa 2011; Gebauer et al. 2011; Gerlai et al. 2000; Rosemberg et al. 2012; Tran and Gerlai 2013). However, the same EtOH concentration increased the time spent in lit area in the light-dark test. High alcohol concentrations can affect motor function and visual perception (Calhoun et al. 2004; Nutt and Peters 1994). In fact, EtOH 1% has been associated to changes in perception (Gerlai et al. 2000; Oliveira et al. 2013). Corroborating these findings, we observed that high EtOH concentrations significantly decreased the PC1 in the novel tank suggesting an impairment in their locomotor profile. Thus, the conflicting data observed in the light-dark test could be related to reduced swimming activity and/or even changes in visual perception when zebrafish are tested in a tank with two different contexts.

TAU alone displayed an anxiolytic profile at the 42 and 400 mg/L without modulating EtOH-induced responses. TAU has anxiolytic-like effects when zebrafish behavior is assessed in the novel tank and in the light-dark tests (Mezzomo et al. 2016). Similar concentration-dependent responses were also previously described, where TAU potentiates EtOH-increased aggression at the lowest and highest concentration, while 150 mg/L displays anti-aggressive action (Fontana et al. 2016). Although TAU changed anxiety-like profiles at the lowest and highest concentration, TAU 150 mg/L prevented anxiolytic-like effects of alcohol at 0.25% and decreased EtOH 1%-induced changes in the light-dark test. All TAU concentrations alone and associated to EtOH also decreased the number of risk assessment episodes. The reduction of risk assessment episodes was previously observed in a fear-related task, where animals showed less caution when exploring the compartment close to a predator (Fontana et al. 2018). Moreover, the lowest and highest concentrations protected against EtOH 1%-induced locomotor impairment. As observed here, mounting evidence shows that TAU does not change locomotion (Fontana et al. 2016; Fontana et al. 2018; Mezzomo et al. 2016; Rosemberg et al. 2012). In zebrafish, TAU pretreatment decreases alcohol content in the brain and prevents EtOH-induced locomotor changes, suggesting a direct influence of EtOH levels in the locomotion (Rosemberg et al. 2012). Additionally, TAU counteracts EtOH-induced anxiogenesis by normalizing the transitions and time spent in top area, only at the highest concentration. TAU is a molecule endogenously produced through cysteine oxidation having important physiological role in living organisms (De Luca et al. 2015; Huxtable 1992; Vitvitsky et al. 2011). Although more studies are needed to fully understand how EtOH and TAU interact, the mechanisms responsible for TAU effects on behavior are mainly associated to the GABAergic and glycinergic agonism (Dzirkale et al. 2011; Frosini et al. 2006), as well as to the antagonism of NMDA receptors (Chan et al. 2014). Thus, the biphasic effect of TAU association may involve complex interactions of molecules with a certain receptor and/or regulatory neurochemical pathways (Calabrese and Baldwin 2001; Fontana et al. 2016; Fontana et al. 2018; Rosemberg et al. 2012).

Interestingly, EtOH did change the responses across time for the time spent in the top area and in the lit area, suggesting that EtOH suppresses the natural decrease of anxiety across time. However, although TAU increased the time spent in the lit area, no significant effect of TAU across time was observed for both anxiety-related tasks (novel tank and light-dark test). Analyzing the interaction of the three factors (time, TAU, and EtOH), a significant effect was observed. Altogether, these data suggest that TAU when associated to EtOH has a positive interaction across time and may attenuate alcohol suppressing the natural zebrafish behavioral response.

PCA analysis of the light-dark supported that both TAU 42 and 400 groups are significantly affecting anxiety-related parameters (PC1). Alcohol anxiolytic-like responses were also mainly observed in the light-dark test through the analysis of the PC1. For the novel tank, the PC1 was related to locomotion and PC2 to anxiety-related parameters. EtOH 1% clearly induced locomotor deficits, and this could be an important factor underlying alcohol-induced changes in anxiety-like phenotypes. TAU 150 counteracted the anxiolytic-like effects of EtOH 0.25%, and all TAU concentrations reduced locomotor and anxiogenic effects of EtOH 1%. Overall, TAU alone and associated to EtOH 0.25% presented a U-shaped profile for anxiolytic-like behavior in the light-dark test, and all TAU concentrations prevented anxiogenic-like responses in the novel tank diving test. These data suggest that the behavioral responses measured here reflect a complex interaction between both molecules.

The association of TAU with EtOH suggests a complex interaction with different behavioral domains (e.g., aggression, anxiety, and locomotion). This association leads to a U-shaped response in scototaxis-, aggression-, and anti-predatory-like responses (Fontana et al. 2016). Importantly, a dose-response is observed for other behavioral domains, such as locomotion, anxiety (novel tank), and social behavior (Fontana et al. 2018). To date, TAU 150 mg/L associated to EtOH has positive effects in the light-dark test and aggressiveness. All TAU concentrations also reduced EtOH-induced locomotor deficits, suggesting a protective role of TAU in this specific behavior. However, concomitant alcohol and 400 mg/L TAU exposure potentiates aggression in the mirror-induced aggression test (Fontana et al. 2016) and decreases the preference for conspecifics (Fontana et al. 2018). Because alcohol disinhibits punished behavior (Fillmore et al. 2008; Johansson and Hansen 2000), concomitant exposure to TAU and EtOH may have more dangerous impact since TAU also decreased the time close to the predator (Fontana et al. 2018).

Although the mechanisms underlying the modulatory role of TAU on EtOH-induced behaviors in zebrafish are still unclear, the complex behavioral effects observed in the presence of both molecules reinforce the involvement of different neurotransmitter systems. Further studies are needed to investigate how TAU and EtOH interact each other. Importantly, the dissociable patterns of behavior observed for the light-dark vs novel tank tests were previously reported (Maximino et al. 2010b; Blaser and Rosemberg 2012), suggesting that both tests are complementary rather than interchangeable and involve different motivational aspects.

## Conclusions

In summary, our novel findings demonstrate that TAU shows a biphasic profile when co-exposed with EtOH, where low and high TAU concentrations decrease anxiety and do not prevent alcohol-mediated anxiolysis. Meanwhile, the intermediate TAU concentration did not change anxiety-related parameters and has a protective effect against EtOH 0.25%-induced behavioral changes. Moreover, all TAU concentrations prevented against sedative effects of alcohol by decreasing anxiogenic-like behavior and reducing locomotor deficits. Overall, TAU and EtOH concomitant exposure have complex behavioral outcomes against alcohol-induced anxiolytic and anxiogenic-like phenotypes, and care should be taken with their simultaneous use. Furthermore, more studies are necessary to investigate the mechanisms underlining how TAU exposure differently modulates EtOH-induced anxiolytic and anxiogenic phenotypes.
